# Surveillance and monitoring of antimicrobial resistance: limitations and lessons from the GRAM project

**DOI:** 10.1186/s12916-019-1412-8

**Published:** 2019-09-20

**Authors:** Jesse Schnall, Arjun Rajkhowa, Kevin Ikuta, Puja Rao, Catrin E. Moore

**Affiliations:** 1 Monash University Clayton Campus, Clayton, Victoria 3800 Australia; 2 The Peter Doherty Institute for Infection and Immunity, University of Melbourne, 792 Elizabeth St, Melbourne, Victoria 3000 Australia; 3 Institute for Health Metrics and Evaluation, 2301 5th Ave, Seattle, WA 98121 USA; 4 Nuffield Department of Medicine, Big Data Institute, Li Ka Shing Centre for Health Information and Discovery, Headington, Oxford, OX3 7LF UK

**Keywords:** Antimicrobial resistance, Drug-resistant infections, Antimicrobial drugs, Global health, Global burden of disease, Public health, Anti-infective agents.

## Background

Reliable estimates of the current and future disease burden of antimicrobial resistance (AMR) are essential to combat the global drug-resistant infection crisis [1, 2]. Yet, despite considerable global efforts, our understanding of the burden of resistant infections remains disturbingly sparse [3]. More reliable, detailed and dynamic information is essential to successfully address an apparent rise in resistant infections, enabling policymakers and healthcare providers to implement national AMR action plans, and efficiently allocate resources.

Major, recent efforts to estimate AMR burden include the Global Burden of Disease (GBD) 2016 study [4], the Review on AMR [1], and a 2015 study conducted by the European Centre for Disease Prevention and Control (ECDC) [5]. However, these provide insufficiently precise or comprehensive estimates to inform effective policies. The figure of 700,000 annual deaths from resistant infections often cited from the Review on AMR suffers from methodological limitations and statistical uncertainty. GBD 2016 focuses exclusively on multi-drug-resistant and extensively drug-resistant tuberculosis, and covers only a fraction of global AMR burden. The ECDC publication focuses only on the European Union and European Economic Area, and depends on European Antimicrobial Resistance Surveillance Network (EARS-Net) data.

### Challenges in AMR surveillance

More accurately measuring AMR burden requires a fully described scientific measurement framework [3]. The global scientific community is responding to this call through the Global Research on Antimicrobial Resistance (GRAM) project, led by the University of Washington’s Institute for Health Metrics and Evaluation, and the University of Oxford’s Big Data Institute. GRAM will be the first study to quantify the incidence, prevalence, excess mortality risk, and overall disease burden attributable to key antibiotic resistant bacteria at the global, national and – where possible – sub-national levels. It will enable direct comparison of AMR burden with other global health threats, and provide an invaluable evidence base to guide policy.

The accuracy of AMR burden estimates depends on the quality and availability of input data [3]. Present global surveillance systems remain disconnected and underdeveloped, with recent recommendations from the United Nations highlighting the urgent need to strengthen AMR surveillance globally [2]. Only 70 countries have enrolled in the World Health Organization (WHO)’s Global Antimicrobial Resistance Surveillance System (GLASS); fewer than 50 reported AMR rates in its most recent call-out [6]. Data are self-reported, heterogeneous, and based on few isolates from a handful of surveillance sites.

The development of GLASS is a step in the right direction, but it is at an early stage [7]. GLASS limitations reflect broader global data insufficiencies that hinder the quantification of AMR burden; many nations lack the laboratory and data management capacities to support effective surveillance [7]. Microbiology data may be available electronically, but clinical datasets are frequently archived in laboratory books and patient notes, creating barriers to analysis. Electronic data also suffers from entry error caused by staffing constraints and a lack of reporting uniformity.

Necessarily stringent privacy concerns further limit data sharing. Linked data that matches clinical bacteria to patient mortality outcomes, patient information and comorbid conditions, are essential to measure the excess mortality risk of resistant infections – and they are virtually non-existent. In the absence of comprehensive and diverse datasets from various regions and income-bases, GBD estimates rely heavily on modeling, which increases uncertainty. This is particularly concerning in low-income regions, where surveillance data is almost non-existent, access to essential antimicrobials is limited, and substandard antibiotics are widely available [2, 3].

High-income nations face their own challenges. An evaluation of Australia’s AMR response highlighted the need for a more robust animal surveillance system, and better One Health integration [8]. Although data-linking is a priority, the absence of consistent antimicrobial susceptibility testing and reporting standards impedes comparison of subnational datasets. Meanwhile, poor documentation and passive extraction methods limit the utility of community antibiotic prescribing data. Australia is one of many countries yet to contribute surveillance data to GLASS.

### Global surveillance initiatives

Despite its shortcomings, Australia’s model of integrating AMR surveillance into system-wide risk-mitigation strategies may offer valuable lessons. The national Antimicrobial Use and Resistance in Australia network has identified alarming trends in resistance and antimicrobial use (AMU) [9]. Surveillance initiatives have gained jurisdictional and national support, with whole genome sequencing identifying the spread of specific resistant bacteria and resistance-causing genes.

AMU surveillance initiatives in Australian tertiary and aged care facilities have facilitated the inclusion of antimicrobial stewardship standards within national medication safety and quality frameworks [9]. Overuse and misuse of antimicrobials is a considerable precursor to increasing resistance; to successfully limit AMR, behavior change strategies must be incorporated into policy schemes. While methodological barriers exist to data integration, efforts to embed AMU surveillance in overarching monitoring frameworks must continue.

Better surveillance infrastructure requires ongoing funding and support from key donors and policymakers. The UK’s £265 million Fleming Fund for surveillance initiatives in low and middle-income countries is a welcome display of global leadership [1]. Likewise, the Australian Government has made $300 million AUD available to address regional infectious disease threats, with AMR earmarked as a key concern [10]. Such funding commitments should be used to support surveillance infrastructure-building efforts, both domestically and in vulnerable regions.

Regional capacity-building must be part of a broad, coordinated international response. Governing bodies must assist countries to build AMR surveillance systems, while supporting the development and funding of national action plans in fragile states – currently, only one-fifth of national action plans are properly funded [7]. To effectively inform the burden of AMR, surveillance systems must coordinate comprehensive, globally consistent datasets through a One Health approach. Mechanisms facilitating increased data sharing, such as WHO’s GLASS initiative, must be supported. Improved public–private collaborations and open data sharing are essential to this goal, enabling greater utilization of vital private sector data.

## Conclusion

Without a more concerted and collaborative global response, the disastrous forecast made in the Review on AMR of 10 million annual deaths from resistant infections by 2050 might eventuate. Initiatives that enhance monitoring capacity for resistant infections, and bolster the accuracy of AMR burden estimates, will provide essential information to guide policy and prevent a calamity of this scale.

## Data Availability

Not applicable.
